# Supplementary material for: Phytopathogenic Curtobacterium flaccumfaciens Strains Circulating on Leguminous Plants, Alternative Hosts and Weeds in Russia
Source: Plants (Basel). 2024 Feb 28;13(5):667. doi: 10.3390/plants13050667 (PMC10934172; doi:10.3390/plants13050667)
Supplement: Supplementary file 1 [file plants-13-00667-s001.zip › Figure S1.pdf]

plants

MDPI

Supplementary Material

Phytopathogenic *Curtobacterium flaccumfaciens* strains circulating on leguminous plants, alternative hosts and weeds in Russia

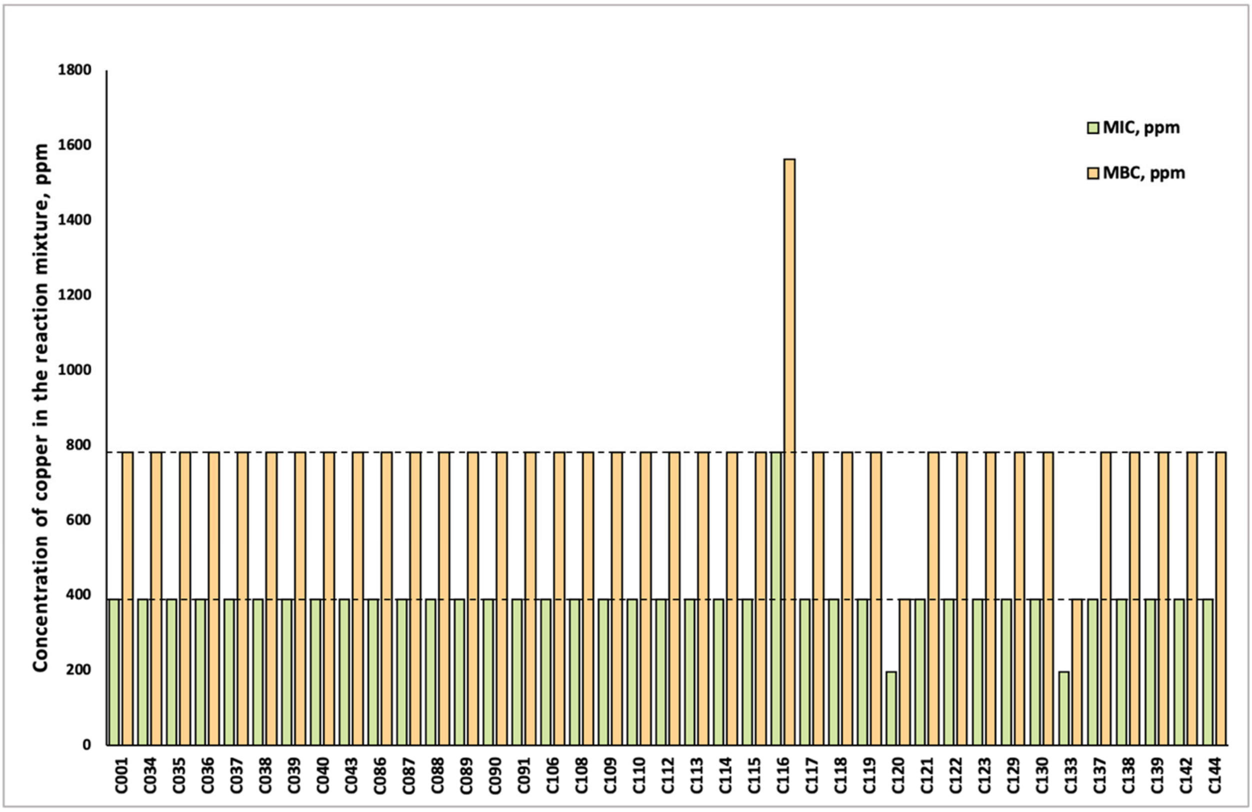

Figure S1. MIC and MBC values for copper dihydroxide against strains of *Curtobacterium flaccumfaciens* measured by the liquid dilution method YD. The values in the boxes represent the respective mean value of four independent experiments. Dashed dividing lines separate susceptible (lower lines) and resistant (upper lines) Cf strains.
