# Supplementary material for: Phytopathogenic Curtobacterium flaccumfaciens Strains Circulating on Leguminous Plants, Alternative Hosts and Weeds in Russia
Source: Plants (Basel). 2024 Feb 28;13(5):667. doi: 10.3390/plants13050667 (PMC10934172; doi:10.3390/plants13050667)
Supplement: Supplementary file 1 [file plants-13-00667-s001.zip › Table S1.pdf]

plants

MDPI

Supplementary Material

Phytopathogenic *Curtobacterium flaccumfaciens* strains circulating on leguminous plants, alternative hosts and weeds in Russia

Table S1. Bacteriological characteristics of strains isolated in this study. Y – yellow, LY – light yellow, «+» - positive, «-» - negative, «+/-» - weak positive.

№	Strain	Curtobacterium sp. PCR	Cff PCR assay	Colour on NBY	Gram stain	Catalase	Cytochrom-c-oxydase	Urease	Indole	Gelatin hydrolysis	Casein hydrolysis	Glucose o/f test - O	Glucose o/f test - F	Acid from mannose	Acid from maltose	Acid from inositol	Acid from inulin	Acid from erythriol
EPPO standard		+	+	Y	+	+	-	-	-	+	+	+	-	+	+	+	-	-
Pathogenic strains from cultivated plants																		
1	C034	+	+	Y	+	+	-	-	-	+	+	+	-	+	+	+/-	+/-	-
2	C035	+	+	Y	+	+	-	-	-	+	+	+	-	+	+	+/-	+/-	-
3	C036	+	+	Y	+	+	-	-	-	+	+	+	-	+	+	+/-	+/-	-
4	C037	+	+	Y	+	+	-	-	-	+	+	+	-	+	+	+/-	+/-	-
5	C038	+	+	Y	+	+	-	-	-	+	+	+	-	+	+	+/-	+/-	-
6	C039	+	+	Y	+	+	-	-	-	+	+	+	-	+	+	+/-	+/-	-
7	C040	+	+	Y	+	+	-	-	-	+	+	+	-	+	+	+/-	+/-	-
8	C043	+	+	Y	+	+	-	-	-	+/-	+	+	-	+	+	+/-	+/-	-
9	C086	+	+	LY	+	+	-	-	-	+	+	+	-	+	+	+/-	+	-
10	C087	+	+	LY	+	+	-	-	-	+	+	+	-	+	+	+/-	+/-	-
11	C088	+	+	LY	+	+	-	-	-	+	+	+	-	+	+	+/-	+/-	-
12	C089	+	+	LY	+	+	-	-	-	+	+	+	-	+	+	+	+/-	-
13	C090	+	+	LY	+	+	-	-	-	+	+	+	-	+	+	+	+/-	-
14	C091	+	+	LY	+	+	-	-	-	+	+	+	-	+	+	+	+/-	-
15	C137	+	+	LY	+	+	-	-	-	+/-	+	+	-	+	-	+	+	-
16	C138	+	+	LY	+	+	-	-	-	+/-	+	+	-	+	-	+	+/-	-
17	C139	+	+	LY	+	+	-	-	-	+	+	+	-	+	-	+	+/-	-
18	C142	+	+	LY	+	+	-	-	-	+	+	+	-	+	-	+	+/-	-
19	C144	+	+	LY	+	+	-	-	-	+	+	+	-	+	-	+	+/-	-
Pathogenic strains from wild plants																		
20	C108	+	+	LY	+	+	-	-	-	+/-	+	+	-	+	+	+	-	-

№	Strain	Curtobacterium sp. PCR	Cff PCR assay	Colour on NBY	Gram stain	Catalase	Cytochrom-c-oxydase	Urease	Indole	Gelatin hydrolysis	Casein hydrolysis	Glucose o/f test - O	Glucose o/f test - F	Acid from mannose	Acid from maltose	Acid from inositol	Acid from inulin	Acid from erythriol
21	C109	+	+	LY	+	+	-	-	-	+	+	+	-	+	+	+	-	-
22	C110	+	+	LY	+	+	-	-	-	+/-	+	+	-	+	+	+/-	+/-	-
23	C112	+	+	LY	+	+	-	-	-	+/-	+	+	-	+	+/-	+	+/-	-
24	C113	+	+	LY	+	+	-	-	-	+/-	+	+	-	+	+/-	+	+/-	-
25	C114	+	+	LY	+	+	-	-	-	+	+	+	-	+	+	+	+/-	-
26	C115	+	+	LY	+	+	-	-	-	+	+	+	-	+	+	+	+/-	-
27	C116	+	+	LY	+	+	-	-	-	+	+	+	-	+	+	+	+/-	-
28	C117	+	+	LY	+	+	-	-	-	+	+	+	-	+	+	+	+/-	-
29	C118	+	+	LY	+	+	-	-	-	+	+	+	-	+	+	+	+/-	-
30	C122	+	+	LY	+	+	-	-	-	+	+	+	-	+	+	+	+/-	-
31	C123	+	+	Y	+	+	-	-	-	+/-	+	+	-	+	+/-	+	+	-
32	C129	+	+	Y	+	+	-	-	-	+	+	+	-	+	+	+	+	-
33	C130	+	+	Y	+	+	-	-	-	+	+	+	-	+	+	+	+/-	-
Reference strains																		
34	C001	+	+	Y	+	+	-	-	-	+	+	+	-	+	+	+	+/-	-
35	C106	+	+	Y	+	+	-	-	-	+	+	+	-	+	+	+/-	+	-
36	C119	+	+	LY	+	+	-	-	-	+/-	+	+	-	+	+	+	+/-	-
37	C120	+	+	LY	+	+	-	-	-	+/-	+	+	-	+	+	+	+/-	-
38	C121	+	+	LY	+	+	-	-	-	+	+	+	-	+	+	+	+/-	-
39	C133	+	+	LY	+	+	-	-	-	+	+	+	-	+	-	+	+	-
