# Supplementary material for: Phytopathogenic Curtobacterium flaccumfaciens Strains Circulating on Leguminous Plants, Alternative Hosts and Weeds in Russia
Source: Plants (Basel). 2024 Feb 28;13(5):667. doi: 10.3390/plants13050667 (PMC10934172; doi:10.3390/plants13050667)
Supplement: Supplementary file 1 [file plants-13-00667-s001.zip › Table S2.pdf]

plants

MDPI

Supplementary Material

Phytopathogenic *Curtobacterium flaccumfaciens* strains circulating on leguminous plants, alternative hosts and weeds in Russia

Table S2. Distribution of statistical groups according to the Duncan criterion according to the values of the affected area of the leaf after the inoculation of soybeans and common beans with *Cf* strains. Values within columns marked by different letters indicate a significant difference, using Duncan's criterion, p=0.05.

№	Strain name	Average values of the leaf lesion area	Groups by criterion Duncan	Strain name	Average values of the leaf lesion area	Groups by criterion Duncan
		after artificial inoculation of soybean cv. Kasatka			after artificial inoculation of common bean cv. Purpurnaya Koroleva	
1	C043	0	a	C043	0	a
2	C115	0	a	C115	0	a
3	C118	0	a	C118	0	a
4	C137	0	a	C137	0	a
5	C091	0,87	b	C090	0,77	b
6	C133	0,88	b	C119	0,92	bc
7	C119	1,11	b	C108	0,93	bc
8	C116	1,76	de	C039	0,96	bc
9	C039	1,77	de	C133	1,05	bc
10	C121	1,77	de	C038	1,06	bc
11	C034	1,81	dec	C091	1,06	bc
12	C108	1,99	efg	C116	1,08	bc
13	C038	2,02	efg	C087	1,09	bc
14	C106	2,06	fg	C121	1,17	bc
15	C110	2,11	g	C112	2,04	def
16	C138	2,72	h	C037	2,14	ef
17	C130	2,83	h	C106	2,16	ef
18	C122	2,85	h	C001	2,17	ef
19	C129	2,89	h	C113	2,2	efg
20	C109	2,94	h	C109	2,48	fgh
21	C035	3,65	i	C120	2,83	hi
22	C001	3,74	i	C114	2,89	hij
23	C037	3,74	i	C035	2,91	hij

№	Strain name	Average values of the leaf lesion area	Groups by criterion Duncan	Strain name	Average values of the leaf lesion area	Groups by criterion Duncan
		after artificial inoculation of soybean cv. Kasatka			after artificial inoculation of common bean cv. Purpurnaya Koroleva	
24	C144	3,81	i	C110	2,91	hij
25	C139	3,82	i	C138	3,3	jk
26	C036	4,63	j	C036	3,49	kl
27	C040	4,66	j	C040	3,54	klm
28	C114	4,74	j	C129	3,76	lmn
29	C120	4,74	j	C144	3,76	lmn
30	C123	4,78	j	C139	3,85	lmn
31	C087	4,8	j	C123	3,96	mn
32	C117	4,82	j	C122	3,97	mn
33	C086	4,87	j	C117	4	n
34	C112	4,87	j	C034	4,06	n
35	C113	4,93	j	C130	4,18	n
36	C090	5,5	k	C142	4,82	o
37	C089	6,45	k	C088	5,24	d
38	C142	6,84	l	C086	5,5	p
39	C088	7,07	l	C089	6,78	q
